# Gambogic acid inhibits thioredoxin activity and induces ROS-mediated cell death in castration-resistant prostate cancer

**DOI:** 10.18632/oncotarget.20424

**Published:** 2017-08-24

**Authors:** Hong Pan, Keith H. Jansson, Michael L. Beshiri, JuanJuan Yin, Lei Fang, Supreet Agarwal, Holly Nguyen, Eva Corey, Ying Zhang, Jie Liu, HuiTing Fan, HongSheng Lin, Kathleen Kelly

**Affiliations:** ^1^ Laboratory of Cancer, Guang’anmen Hospital, China Academy of Chinese Medicine Sciences, Beijing, China; ^2^ Clinical Medical College, Beijing University of Chinese Medicine, Beijing, China; ^3^ Laboratory of Genitourinary Cancer Pathogenesis, Center for Cancer Research, National Cancer Institute, National Institutes of Health, Bethesda, Maryland, USA; ^4^ Department of Urology, University of Washington, Seattle, Washington, USA

**Keywords:** gambogic acid, CRPC, ROS, thioredoxin, organoids

## Abstract

Advanced prostate cancer (PrCa) is treated with androgen deprivation therapy, and although there is usually a significant initial response, recurrence arises as castrate resistant prostate cancer (CRPC). New approaches are needed to treat this genetically heterogeneous, phenotypically plastic disease. CRPC with combined homozygous alterations to *PTEN* and *TP53* comprise about 30% of clinical samples. We screened eleven traditional Chinese medicines against a panel of androgen-independent *Pten/Tp53* null PrCa-derived cell lines and identified gambogic acid (GA) as a highly potent growth inhibitor. Mechanistic analyses revealed that GA disrupted cellular redox homeostasis, observed as elevated reactive oxygen species (ROS), leading to apoptotic and ferroptotic death. Consistent with this, we determined that GA inhibited thioredoxin, a necessary component of cellular anti-oxidative, protein-reducing activity. In other clinically relevant models, GA displayed submicromolar, growth inhibitory activity against a number of genomically-representative, CRPC patient derived xenograft organoid cultures. Inhibition of ROS with N-acetyl-cysteine partially reversed growth inhibition in CRPC organoids, demonstrating ROS imbalance and implying that GA may have additional mechanisms of action. These data suggest that redox imbalances initiated by GA may be useful, especially in combination therapies, for treating the heterogeneity and plasticity that contributes to the therapeutic resistance of CRPC.

## INTRODUCTION

PrCa is the most diagnosed and third most deadly cancer among men in Western industrialized nations [1]. Most prostate cancer-related deaths are attributable to metastatic spread. Metastatic PrCa is treated with androgen deprivation therapy (ADT) because most prostate cancers require androgen receptor (AR) signaling to maintain growth and viability. However, almost all men with metastatic PrCa develop resistance to ADT, referred to as metastatic castrate-resistant prostate cancer (mCRPC) [2, 3]. Acquired resistance to ADT is mainly the consequence of genetic alterations to components of the AR signaling pathway that restore activity even with castrate levels of androgens [4, 5]. Following the development of resistance to AR pathway inhibitors, relatively few treatments currently exist. Some patients may temporarily respond to docetaxel [6], or to the PARP inhibitor olaparib, in the case of those ~15% of patients with homologous recombination deficiency [7]. There is a need for additional therapeutic approaches to deter mCRPC progression.

Underlying drivers of aggressive PrCa growth are heterogeneous and include aberrations in a variety of pathways and downstream functions [5]. Among these, genetic modifications to *PTEN* and *TP53* are the most commonly observed mutations, after the AR pathway, associated with mCRPC. The loss or mutation of *PTEN,* a negative regulator of the PI3K/AKT pathway, occurs in about 20% of primary prostate cancers and is enriched to about 40% in CRPC [4, 5, 8]. In parallel with *PTEN* mutations, *TP53* genomic modifications are significantly increased (from about 10 to 50%) in mCRPC relative to primary PC, with co-occurring *PTEN/TP*53 mutations observed in about 30-35% of mCRPC [4, 5, 8]. Subclonal analyses of heterogeneous primary tumors and metastases suggest that acquisition of *TP*53 mutations is linked with increased metastatic potential [9, 10].

mCRPC encompasses a continuum of phenotypes from well-differentiated carcinomas to poorly differentiated tumors that exhibit lineage plasticity [11, 12]. For aggressive cancers such as mCRPC that are characterized by genomic heterogeneity and display a potential for plasticity, one approach to treatment is to identify drugs that target commonly occurring physiological and metabolic vulnerabilities across of spectrum of genotypes and phenotypes. Natural products, such as those used in traditional Chinese medicine (TCM), are one source of bioactive anti-cancer small molecules [13]. In screening a TCM library, we identified gambogic acid (GA) as having a potent activity for a number of phenotypically and genotypically diverse models of advanced PrCa.

GA is a naturally occurring polyprenylated xanthone-based moiety derived from the resin of *Garcinia hanburyi* trees [14]. GA potently inhibits cancer cell proliferation in various cancer cell lines. A variety of indirect effects on signaling pathways and biological functions have been described for GA treatment of individual cancer cell lines [14, 15]. It will be important in evaluating the translational potential of GA to determine the primary biochemical targets and whether there are unifying mechanisms to explain context-dependent anti-proliferative effects. In addition, establishing the generality of efficacy for specific cancer types, including the use of the best predictive human cancer models, is important for the design of future clinical trials evaluating GA.

## RESULTS

### GA is a traditional Chinese medicine with potent activity toward PrCa cells

**Figure 1:**
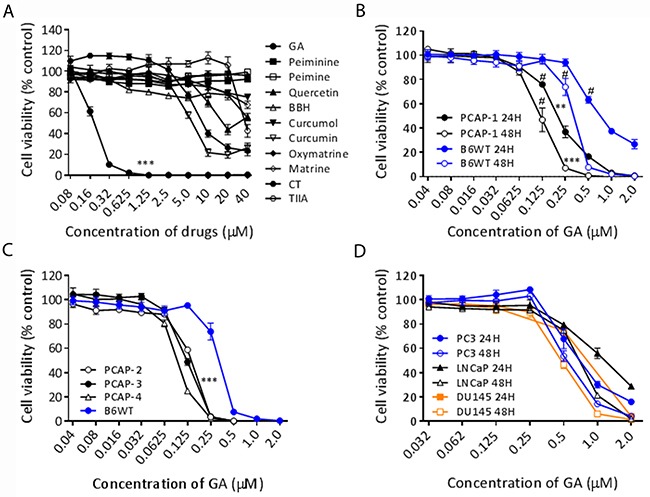
GA inhibited proliferation of prostate cancer cells in a dose- and time-dependent manner **(A)** PCAP-1 cells were treated with the indicated drugs at various concentrations for 48 hours, and cell viability in all panels was determined by MTS assays. ***P<0.001, GA *vs* the other drugs. **(B)** PCAP-1 and B6WT cells were incubated with the indicated concentrations of GA for 24 and 48 hours. ** P<0.01, *** P<0.001, *vs* the B6WT. # minimum GA concentration led to the statistical significance from control. **(C)** PCAP cell lines derived from 3 independent tumors and B6WT cells were incubated with GA for 24 hours. *** P<0.001 *vs* the B6WT **(D)** PC3, LNCaP and DU145 human prostate cancer cell lines were incubated with the indicated concentrations of GA for 24 and 48 hours. The results shown here are the average of three independent experiments. Data are Mean ± SE.

We derived several cell lines from independent PbCre4; *Pten^fl/fl^Tp53^fl/fl^* prostate adenocarcinoma tumors, designated PCAP 1-8 (Prostate Cancer Adenocarcinoma *Pten/Tp53* null). These cell lines are KRT8^+^/AR^+^ and form tumors upon subcutaneous injection. Consistent with the castration insensitive phenotype of the genetically engineered mouse model [16, 17], the cell lines grew in the absence of serum and DHT, demonstrating an aggressive androgen independent phenotype. To uncover novel therapeutic vulnerabilities in highly aggressive PrCa cells, we screened 11 Chinese medicine extractives (Table 1) which have demonstrated anti-cancer activity in various cancer types, including prostate cancer [18–20]. Preliminary screening of the 11 compounds on PCAP-1 cells was carried out to determine efficacy and potency. Following incubation with different compounds for 48 hours, GA demonstrated sub-micromolar activity (IC_50_ = 185nM) which was 50 times more potent than the next most active compounds, curcumin and tanshinone (CT) (Figure 1A). GA inhibited tumor cell proliferation in a concentration- and time- dependent manner while nontumorigenic luminal epithelial cells derived from a normal C57BL/6 prostate (B6WT, KRT8^+^/AR^low^ >80%, KRT8^+^/KRT5^+^ <20%) were relatively resistant to GA (Figure 1B). Additional PCAP lines showed similar responses to GA as PCAP-1, demonstrating a generalizable vulnerability (Figure 1C). To further describe the anti-proliferative activity of GA with respect to PrCa, various human prostate cancer cell lines were assayed and demonstrated sub-micromolar sensitivity (Figure 1D). Taken together, these data reveal GA as a previously unappreciated, potent, anti-proliferative agent for advanced PrCa.

**Table 1:** Traditional Chinese medicine compounds

Name (Abbreviations)	TCM resource (Latin)	IC_50_
Gambogic acid	Garcinia hanburyi.	0.1μM
Peiminine	Fritillaria thunbergii.	N/A
Peimine	Fritillaria thunbergii.	N/A
Quercetin	Bupleurum chinense DC.	8.7μM
Berberine hydrochloride (BBH)	Coptis chinensis Franch.	N/A
Curcumol	Curcuma longa L.	N/A
Curcumin	Curcuma longa L.	5.8μM
Oxymatrine	Sophora flavescens.	N/A
Matrine	Sophora flavescens.	N/A
Crypto-tanshinone (CT)	Salvia miltiorrhiza Bge.	8.1μM
Tanshinone IIA (T IIA)	Salvia miltiorrhiza Bge.	N/A

The natural products source and IC_50_ towards PCAP-1 for various TCM compounds are shown. N/A indicates that the compound inhibited less than 50% cell growth within the maximum concentration of 40μM.

### GA inhibits prostate cancer stem/progenitor cells and patient-derived organoids

**Figure 2:**
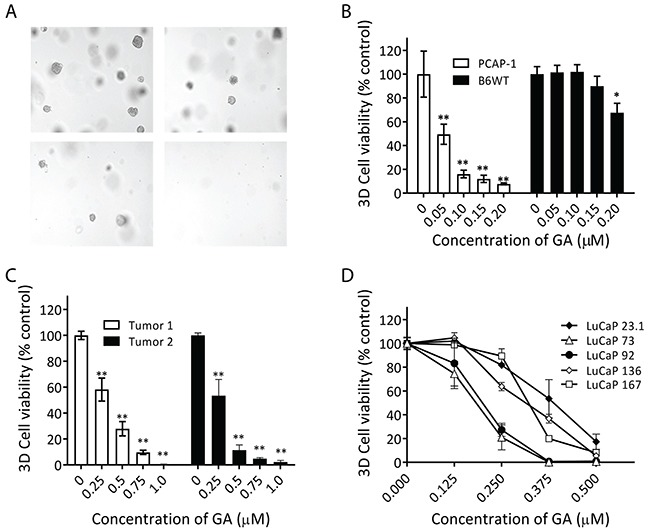
GA inhibited prostate cancer organoid growth **(A-B)** PCAP-1 cells grown in 3D were treated with the indicated concentrations of GA for 7 days. **(C)** Primary PbCr4;*Pten^fl/fl^Tp53^fl/fl^* single cell suspensions were cultured as organoids and incubated with the indicated concentration of GA for 7 days. **(D)** Organoid cultures derived from the indicated LuCaP PDXs were incubated with GA, which was changed every other day for 2 weeks. All results shown are the average of three independent experiments. Data here are expressed as Mean ± SE; *P<0.05, **P<0.01, *vs* the untreated group.

Three-dimensional (3D) organoid culture supports the growth of prostate cancer stem/progenitor cells as well as patient-derived metastatic prostate cancer that normally does not thrive in standard two-dimensional (2D) tissue culture [21, 22]. PCAP and B6WT cell lines initiate organoid growth at a frequency of 10-20 and 1-2%, respectively, demonstrating populations of clonogenic stem/progenitor cells. Microscopic examination of GA-treated, clonally-derived PCAP-1 organoids showed that GA progressively decreased organoid numbers and size (Figure 2A). In fact, the relative potency of GA for PCAP-1 organoids was 2-3 times greater when compared to 2D cultures, while B6WT derived organoids were significantly less sensitive than PCAP organoids (Figure 2B). To determine the sensitivity of primary tumor stem/progenitor cells we analyzed the effect of GA on organoids derived from the luminal fraction of primary PbCre-4; *Pten^fl/fl^Tp53^fl/fl^* tumors [23]. We again observed sensitivity in the submicromolar range (Figure 2C). Thus, we conclude that self-renewing *Pten/Tp53* null prostate cancer stem/progenitor cells are highly sensitive to GA.

To investigate the generality of CRPC responses to GA, we used various tumors from the genomically-representative LuCaP mCRPC patient-derived xenograft (PDX) cohort [24], analyzed in tumor-derived organoid cultures. Recent analyses of pre-clinical therapeutic trials using PDX cohorts demonstrated a high correlation with genomically-matched patients in clinical trials, emphasizing the utility of PDX models [25]. Importantly, mCRPC organoid growth was generally sensitive to GA in the sub-micromolar range (Figure 2D). Among the PDX's examined, *PTEN* (LuCaP-136) and *TP53* (LuCaPs-136, 92, and 73) altered genotypes are included but do not solely account for sensitivity (Supplementary Table 1) [24, 26]. Taken together, these data demonstrate potency and efficacy of GA for a range of clinically relevant prostate cancer models.

### GA treatment leads to CASP-dependent cell death

**Figure 3:**
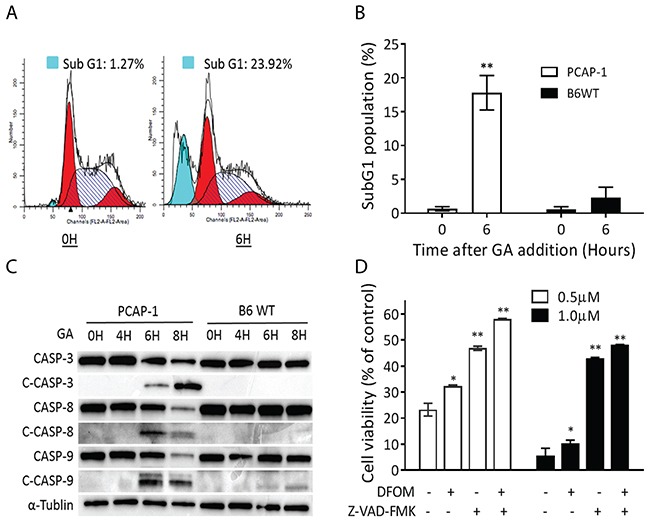
GA induced apoptosis and ferroptosis **(A-B)** PCAP-1 cells treated with 500nM GA for 6 hours were analyzed by flow cytometry, and the percentage of cells demonstrating a sub G1 peak were quantified. **(C)** Western blot analysis of total and cleaved CASP proteins in extracts from GA-treated PCAP-1 cells for the indicated times. **(D)** PCAP-1 cells were treated with GA alone or in combination with either Z-VAD-FMK (20μM) and/or DFOM (100μM) for 24 hours. Cell viability was assessed by an MTS assay. (A) and (C) are representative of three independent experiments. (B) and (D) are the average of three independent experiments. Data here are expressed as Mean ± SE; **P<0.01 vs the untreated group.

To determine whether the growth inhibitory effect of GA in PrCa was attributable to apoptosis, PCAP-1 cells were treated with GA for 6 hours and subsequently stained with propidium iodide (PI) and analyzed by flow cytometry (Figure 3A). Quantification of the sub-G1 population in PCAP1 and B6WT cells indicated that cell growth inhibition was rapid, specific to tumor cells, and contained a significant component of apoptotic cells (Figure 3B).

Apoptosis, which is mediated by both extrinsic cell surface receptor pathways and an intrinsic mitochondrial pathway converge on cysteine-aspartic proteases (CASP) activation, including CASP-3, 8, and 9 [27]. Consistent with the PI analysis, we observed rapid CASP cleavage, an indicator of apoptosis activation, following GA treatment of PCAP-1 but not B6WT cells (Figure 3C). Furthermore, because CASP activation can be the indirect result of cell death initiated by other mechanisms, we evaluated cell growth in the presence of the CASP inhibitor, Z-VAD-FMK. In addition, we also considered ferroptosis, a death mechanism resulting from iron-dependent oxidation injury [28]. As shown in Figure 3D, GA-initiated inhibition of cellular metabolism was partially reversible with either Z-VAD-FMK or with deferoxamine mesylate salt (DFOM), an iron chelator, and both inhibitors together improved cellular viability in the presence of GA. These results suggest that GA initiates CASP-dependent death of PCAP-1 cells and that both iron-dependent oxidative injury and direct CASP activation contribute.

### GA induced rapid ROS accumulation and mitochondrial dysfunction

**Figure 4:**
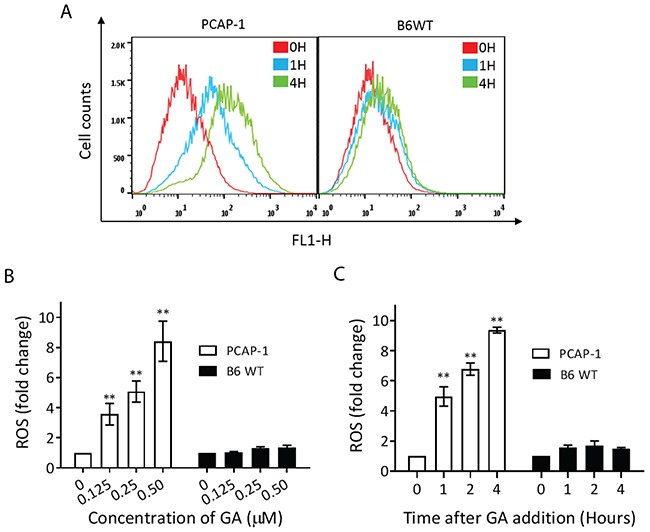
GA induced ROS accumulation preferentially in the tumor as compared to normal prostate epithelial cells **(A)** Intracellular ROS accumulation was measured by flow cytometry with CM-H2DCFDA in PCAP-1 and B6WT cells treated with 500nM GA. **(B-C)** Intracellular ROS accumulation as measured in (A) was calculated and represented as the fold change relative to untreated cells. All results shown are the average of three independent experiments. Data here are expressed as Mean ± SE; **P<0.01 *vs* the control group.

GA induced apoptosis and ferroptosis, both of which can result from excess intracellular ROS [29, 30]. Therefore, we examined PCAP-1 and B6WT cells by flow cytometry using the ROS-sensitive probe, CM-H2DCFDA, at various times after GA incubation (Figure 4A). GA treatment of PCAP-1 but not B6WT cells strongly increased ROS levels in a concentration range that correlated with cell killing (Figure 4B and 1B). Importantly, time course analysis showed rapid increases in ROS levels by 1 hour, suggesting that ROS induction is an early event following GA treatment (Figure 4C).

**Figure 5:**
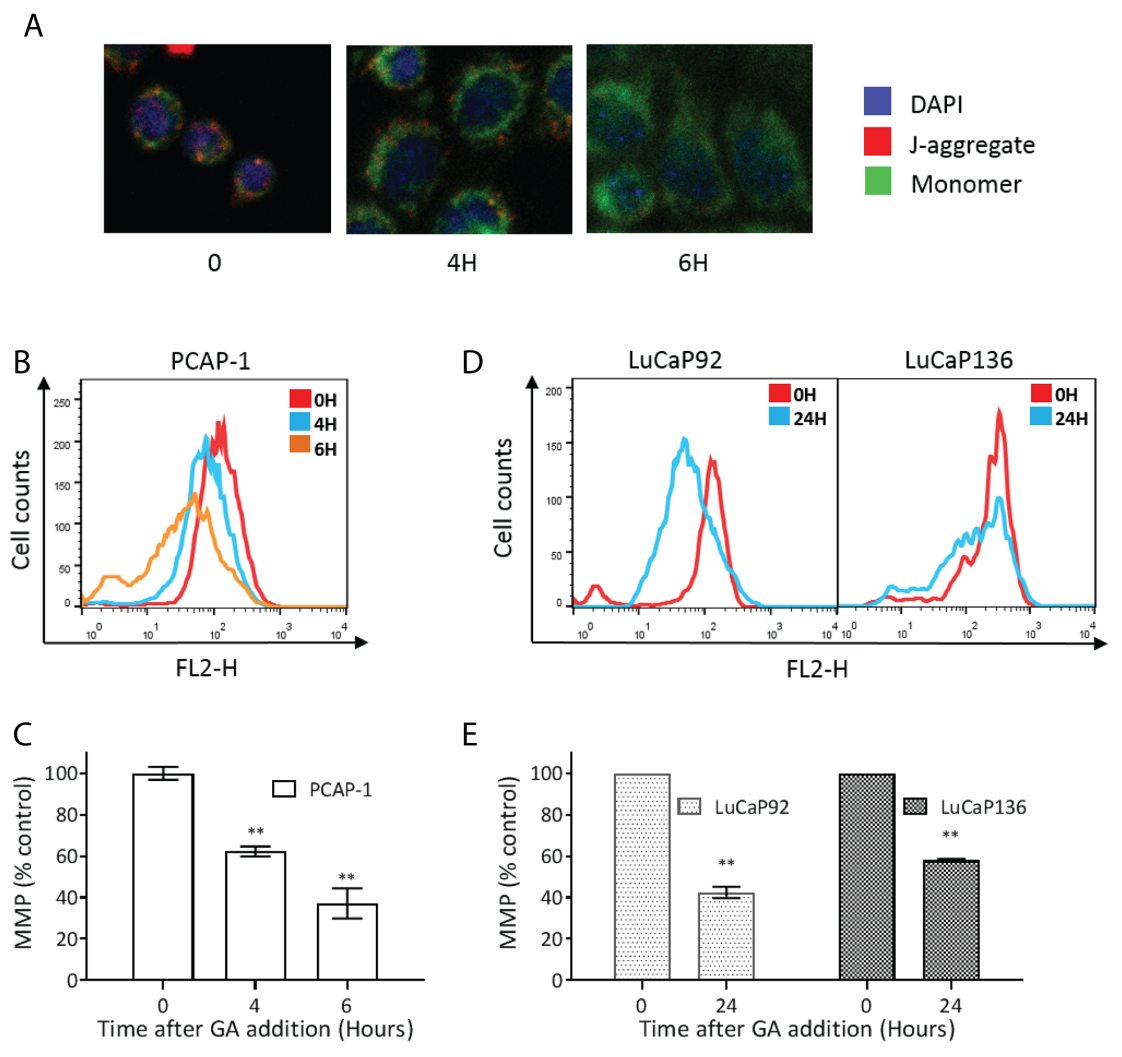
GA induced mitochondrial dysfunction in mouse and human prostate cancer cells **(A)** GA-induced changes to MMP were detected in PCAP-1 cells incubated with 500nM GA observed as a shift in the JC-1 probe from red to green. **(B-E)** GA treated cells were assayed for TMRM fluorescence by flow cytometry, and the differences in emission spectra were calculated as the percentage overlapping the peak of untreated cells. All results shown are an average of three independent experiments. Data here are expressed as Mean ± SE; **P<0.01 v*s* the untreated group.

One potential outcome of excessive ROS is mitochondrial dysfunction, leading to loss of mitochondrial membrane potential (MMP) [31]. We observed a shift of red to green fluorescence of the JC-1 molecular probe in PCAP-1 cells treated with GA for 4 hours (Figure 5A), indicating decreased MMP. Consistent with this, flow cytometry using the TMRM probe demonstrated that GA treatment showed increasing loss of membrane polarity at 4 and 6 hours in PCAP-1 cells (Figures 5B and 5C). In addition, we observed similar evidence of mitochondrial dysfunction in GA-treated LuCaP92 and LuCaP136 derived organoid cultures (Figures 5D and 5E). Collectively, these results support the conclusion that GA treatment most likely induced mitochondrial dependent apoptosis in the prostate cancer cells.

### Formation of ROS is causal for anti-proliferative, pro-apoptotic effects of GA in prostate cancer cells

**Figure 6:**
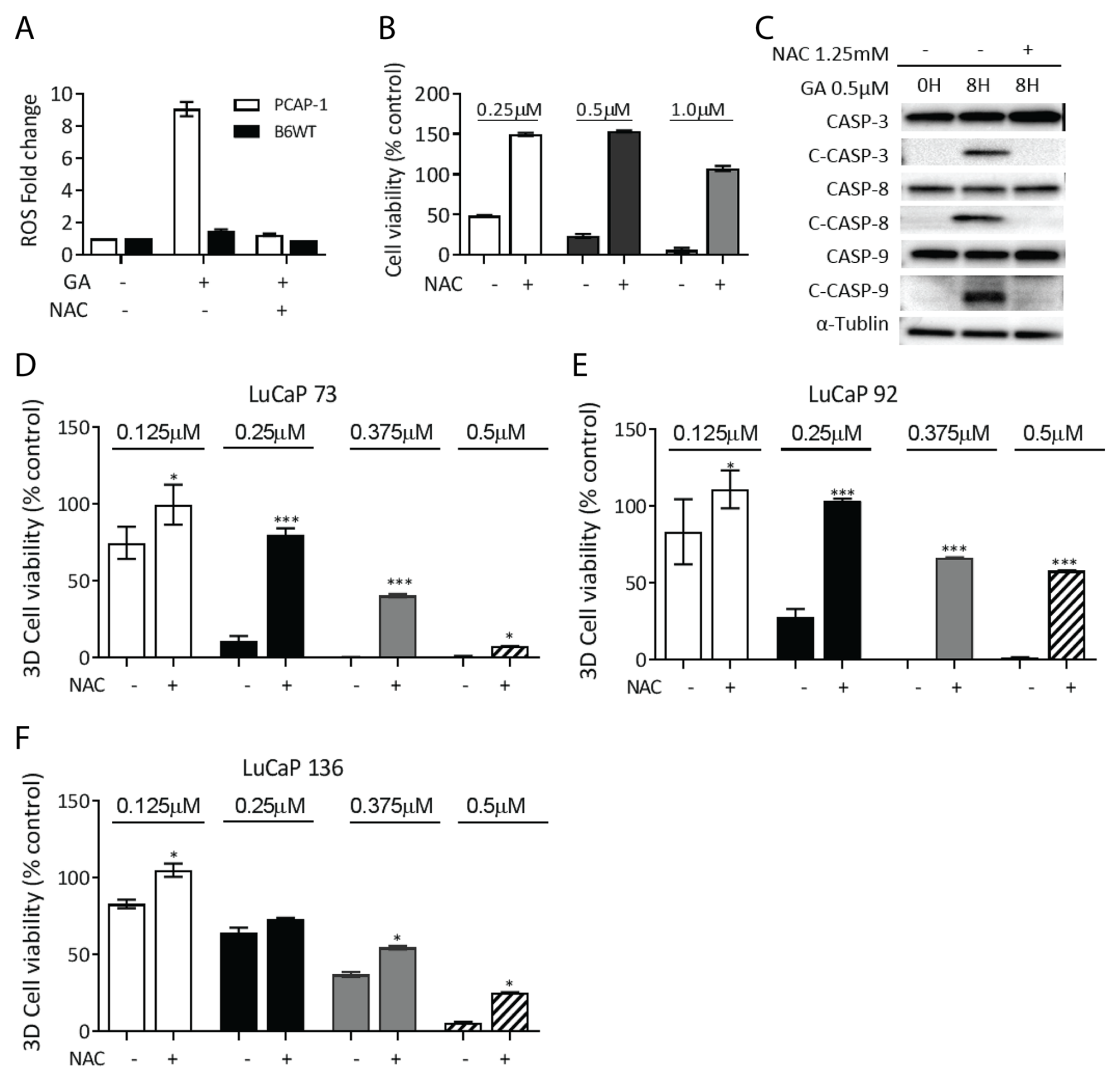
Anti-oxidant co-incubation reverses GA-mediated growth inhibition **(A)** ROS accumulation in PCAP-1 and B6WT cells was determined 6 hours after GA treatment in the presence and absence of NAC. **(B-C)** PCAP-1 cells were treated with GA in the presence and absence of NAC, proliferation (B) and CASP cleavage (C) were analyzed after 24 hours. **(D-E)** PDX-derived organoids were treated with the indicated concentrations of GA in the presence and absence of NAC and growth was measured after 2 weeks. All results shown are representative of at least three independent experiments. Data here are expressed as Mean ± SE; *P<0.05, **P<0.01 *vs* the control group.

The rapid accumulation of ROS following GA treatment suggested that a disruption of redox balance may be a major mechanism leading to GA-mediated anti-proliferative activity. To further investigate whether ROS formation is causal in GA induced cell apoptosis, we treated PCAP-1 cells with GA in the presence of N-acetyl cysteine (NAC), an antioxidant. The presence of NAC inhibited ROS accumulation (Figure 6A), alleviated growth inhibition, even following 1μM GA treatment (Figure 6B), and inhibited GA-induced CASP cleavage (Figure 6C). These results indicate that ROS formation represents the main mechanism of GA induced cell apoptosis in PCAP-1 cells. In addition, NAC partially protected LuCaP73, LuCaP92, and LuCaP136 organoids from GA-mediated growth inhibition (Figures 6D, 6E and 6F). These data suggest that ROS accumulation is one significant mechanism of growth inhibition in these genomically-heterogeneous patient-derived models.

### GA inhibits thioredoxin activity

**Figure 7:**
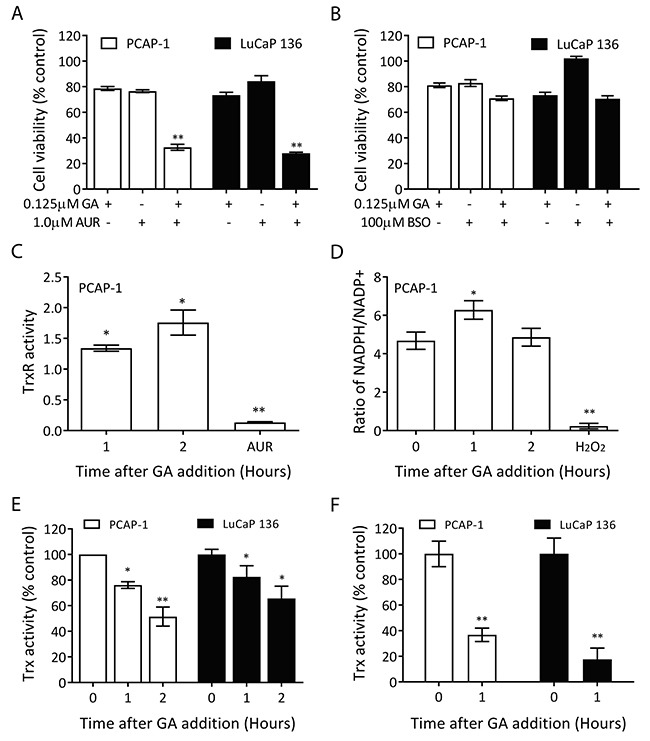
GA inhibits Trx activity **(A-B)** PCAP-1 cells and PDX-136 organoids were treated with GA alone or in combination with AUR 8uM (A) or BSO 100uM (B) for 24 hours (PCAP-1) or 2 weeks (PDX-136) and cellular viability was determined. **(C)** Cellular extracts from PCAP-1 cells incubated with 500nM GA for the indicated times were analyzed for thioredoxin reductase activity, which is expressed as a ratio relative to untreated. AUR treatment was used as a positive control. **(D)** Extracts of PCAP-1 cells incubated with 500nM GA for the indicated times were analyzed for NADPH and NADP+ levels. 1% H_2_O_2_ was used as a positive control. **(E-F)** PCAP-1 cells or PDX-136 organoids were treated with 500nM and 1μM GA, respectively, for the indicated times; extracts were prepared and Trx activity was analyzed without (E) or with (F) the addition of GA to the extract. Results shown here are the average of three independent experiments. *P<0.05, **P<0.01, *vs* the untreated group.

Tumor cells often generate higher basal levels of ROS than normal cells [32], and the rapid accumulation of ROS in GA-treated prostate cancer cells suggested that GA may inhibit a component of the anti-oxidant system. Therefore, we analyzed a variety of inhibitors targeting various components of antioxidant pathways for synergy with GA. Auranofin (AUR), a thioredoxin reductase (TrxR) inhibitor was the one compound that demonstrated additive growth inhibition together with GA when both were combined at sub-thresh hold concentrations (Figure 7A, Supplementary Figure 1A). The specificity of this approach is illustrated by suboptimal concentrations of buthionine sulfoximine (BSO), an inhibitor of the GSH pathway, that did not affect GA efficacy upon combination (Figure 7B, Supplementary Figure 1B). These results suggest that GA may inhibit the thioredoxin (Trx) system, which mainly composes NADPH, TrxR, and Trx.

The effect of GA upon Trx system components was analyzed in PCAP-1 cells. As shown in Figures 7C and 7D, incubation of cells with GA did not reduce, but slightly increased, the TrxR activity and NADPH/NADP^+^ ratio in cells. By comparison, Trx activity was consistently decreased over time in PCAP-1 cells and LuCaP136 organoids following incubation with GA (Figure 7E). In addition, GA addition directly to cellular extracts led to loss of Trx activity (Figure 7F). As NADPH reduces TrxR and TrxR reduces Trx, an increase in NADPH and reduced active TrxR is consistent with a loss of Trx as a substrate for reduction. These data taken together with the observation of cellular ROS accumulation support the conclusion that Trx inhibition is a major activity of GA.

### GA enhanced the anti-tumor effect of docetaxel and enzalutamide

**Figure 8:**
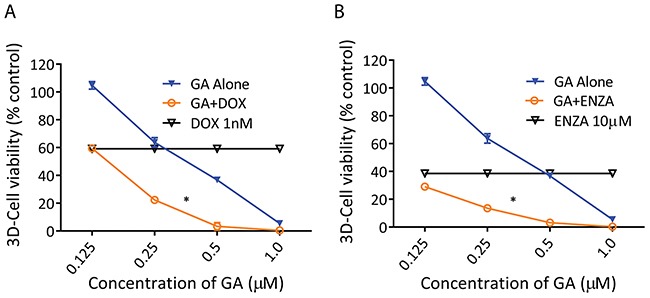
GA enhanced the anticancer activity of docetaxel (DOX) and Enzalutamide (ENZA) on PDX-136 organoids **(A-B)** PDX-136 organoids were treated with the indicated concentrations of GA in the presence of DOX (A) or ENZA (B) for 2 weeks, and the relative cell viability was measured by a CTG-3D assay. *P<0.05 vs GA alone.

Drug combinations often improve efficacy and decrease resistance for the treatment of metastatic cancers. PDX models have proven valuable in predicting patient therapeutic responses [25], and here we used LuCaP136 PDX-derived organoid cultures to evaluate the potential use of GA in combination with standard chemotherapeutic (docetaxel) and anti-androgen endocrine (enzalutamide) therapies for advanced PrCa. As shown in Figure 8, submicromolar concentrations of GA enhanced the cell killing observed for either docetaxel (DOX) or enzalutamide (ENZA). Synergy between drugs occurs when the combination index (CI) is <1 [33]. The CI values calculated by CompuSyn software were mostly <1, indicating that the combination of GA and DOX or ENZA have a synergistic cytotoxic effect in LuCaP136 (Supplementary Table 2, 3). These results suggest that combining GA with the current standard of care therapeutics may improve the fractional response.

## DISCUSSION

We identified GA among several traditional Chinese medicines as a potent therapeutic for inhibiting the proliferation of aggressive prostate cancer. Genetically-engineered mouse *Pten/Tp53* null-derived prostate luminal progenitor cell lines and primary organoid cultures, which model an AR-independent stage of disease [16, 17], were highly sensitive to GA-mediated growth inhibition. In addition, GA was active in a variety of heterogeneous and clinically-relevant CRPC models, assayed in 3D organoid cultures. These LuCaP PDX models contain various genomic driver and acquired resistance mutations, representing human disease phenotypes that are refractory to current treatments [26]. Taken together, these data suggest that GA targets common vulnerabilities across various genotypes and phenotypes present in advanced PrCa.

Mechanistic investigations using PCAP-1 cells revealed that CASP activation and subsequent cell death was attenuated by Z-VAD-FMK and to a lesser degree by iron chelation, suggesting engagement of endogenous apoptosis/ferroptosis pathways [27, 28, 30]. Further, the NAC mediated reversion of rapid ROS elevation and subsequent CASP activation in PCAP-1 but not B6WT cells demonstrated a central role for ROS in mediating GA activity. Because cancer cells often demonstrate metabolism leading to heightened ROS levels that sensitize them to changes in redox balance [32], we analyzed ROS generating and neutralizing pathways as targets of GA. Specific synergistic anti-proliferative effects of GA in combination with AUR suggested that GA targets the thioredoxin system [34]. We observed inhibition of Trx activity in PCAP-1 cells and human LuCaP organoids treated with GA as well as direct inhibition of Trx in cellular extracts with added GA. The activity of upstream components, NADPH and TrxR, in the Trx pathway were slightly increased by GA treatment, consistent with the loss of Trx as an oxidizing substrate. In support of this, a previous report has shown the covalent modification and inhibition of purified, recombinant Trx by GA [35].

Trx reverses protein oxidation directly and also supports hydrogenperoxide clearance by reactivating peroxiredoxins [34]. Decreased peroxiredoxin activity is consistent with the contribution of ferroptosis to GA-mediated cell death [28]. Although NAC completely reversed the anti-proliferative effects of GA in PCAP-1 cells, NAC only partially inhibited cell death in GA-treated human LuCaPs models. One possibility is that there are critical targets in the human LuCaP models that are effected by GA-modified Trx, even in a reducing environment. For example, Trx directly binds and regulates the reduction of a number of transcription factors, including steroid receptors [36, 37]. Unlike the *Pten/Tp*53 null mouse organoids, the adenocarcinoma LuCaPs organoids decrease proliferation in the presence of androgen receptor inhibitors (Beshiri, Agarwal, Jansson, and Kelly, unpublished). Another consideration is that GA has been reported to target additional proteins such as HSP90 [38]. HSP90 is a pleiotropic regulator of multiple signaling pathways and has previously been shown to inhibit PrCa growth, in part by inhibiting AR signaling [39].

In summary, we provide strong evidence that GA is a potent anti-proliferative drug for genomically heterogeneous mCRPC. A potential utility for GA is its combination with other therapeutics currently in use or development to treat this refractory disease state [2, 3]. We present evidence here for GA targeting of Trx, a major function of which is to maintain proper protein redox balance. GA has gained attention as a potential therapeutic in a variety of cancers, although additional in-depth analyses in pre-clinical models are necessary for most cancer types [15]. We speculate that the broad efficacy of GA relates to its pleiotropic effects resulting from targeting a common cancer vulnerability, oxidative stress, and multiple protein functions.

## MATERIALS AND METHODS

### Cell culture and reagents

Four luminal prostate epithelial cell lines were derived from independent PbCre4; *Pten^fl/fl^Tp53^fl/fl^* prostate adenocarcinoma tumors, designated PCAP 1-4 (Prostate Cancer Adenocarcinoma *Pten/Tp53* null), and cultured in WIT-P media (Cellaria Biosciences, Cambridge, MA, USA). A normal luminal epithelial prostate cell line (B6WT) was similarly established from a normal C57/BL6 mouse prostate. Human prostate cancer cell lines (PC3, LNCaP, DU145) were originally obtained from ATCC and maintained as described previously. GA, Perminine, Permine, Qurecetin, BBH, Curcumin, Curmunol, Matrine, Oxymatrine, CT, TIIA were purchased from National Institute for Food and Drug Control (Beijing, China). All compounds were prepared as 40mM stocks in DMSO and stored at -20°C in the dark for up to two months. CellTiter-Glo® 96 Aqueous One Solution Cell Proliferation Assay (CTG) kit, Cell Titer-Glo® 3D Cell Viability (CTG-3D) Assay kit and NADPH/NADP Glo assay kit were purchased from Promega (Madison, WI, USA). Antibodies directed against CASP3 (9662), CASP8 (4790), and CASP9 (9508) were obtained from Cell Signaling Co. Ltd (Boston, MA, USA). α-tublin (T8203) antibody was from Sigma (St. Louis, MO, USA). Fluorescent Thioredoxin Activity Assay Kit (20039) and Thioredoxin Reductase Colorimetric Assay Kit (10007892) were purchased from Cayman (Ann Arbor, MI, USA).

### Cell proliferation assay

Mouse cells (PCAPs, B6WT) and human cells (LNCaP, DU145 or PC3) were seeded in 96-well plates at a density of 4,000 cells per well, and incubated at 37°C, 5% CO_2_ for 24 hours to allow for attachment. Cells were treated with the indicated drugs at the indicated concentrations for 24 or 48 hours. DMSO (0.01%) and medium only were used as a vehicle and blank controls, respectively. Cell viability was measured using a CTG assay kit according to the manufacturer's instructions.

### Three dimensional (3D) organoid culture

PCAP and WT cell lines were cultured in 3D as described previously [40] except that WIT-P media was used. Cells were treated 24 hours after seeding. Media was changed every other day for one week. Primary PbCre4; *Pten^fl/fl^Tp53^fl/fl^* luminal cells were cultured as described [23]. LuCaP cells were isolated as single cell suspensions from PDX tumors and cultured as described for metastatic prostate cancer biopsies [21] with the modification that SB202190 was left out of the culture media. We have determined that LuCaP PDX-derived cultures proliferate in organoid culture and maintain their phenotype relative to the originating PDX (Beshiri et al. in preparation). For organoid culture viability assays, cells were seeded at a density of 1,000 (PCAP) and 15,000 (primary prostate and LuCaP) cells per 20μl matrigel/well of a 48-well plate. Following solidification of the matrigel, 0.4 ml of media was added and incubated for 24 hours prior to the addition of drugs. Mouse and LuCaP cells were treated with GA for 1 week and 2 weeks, respectively. Media was changed every 2 days. Cell viability was measured using the CTG-3D assay kit. Briefly, 150μl DMEM was added to each well with 150μl assay reagent and mixed vigorously for 5 minutes to induce cell lysis. Following an additional 25 min incubation at room temperature, 100μl was transferred into a 96-well plate and the luminescent signal was read.

### Cell cycle analysis

To analyze cell cycle distribution, cells were treated with GA (500nM) for the indicated times and subsequently harvested and fixed in 70% ethanol at 4°C overnight. Fixed cells were stained in 5ml PI staining solution containing 0.02mg/ml PI (Thermo Fisher Scientific, Waltham, MA), 0.2mg/ml RNase A and 1% Triton X-100 (v/v) (Sigma, St. Louis, MO) in PBS for 30 min in the dark. Samples were run on the FACS Calibur (BD Biosciences, Heidelberg, Germany), and cell cycle distribution was analyzed by ModFit (Version 3.0).

### Measurement of intracellular reactive oxygen species (ROS)

Intracellular ROS was assessed using the ROS-sensitive dye 5-(and-6)-chloromethyl-2,7’-dichlorodihydrofluorescein diacetate (CM-H2DCFDA) (Invitrogen, Carlsbad, CA). CM-H2DCFDA is non-fluorescent until oxidized in the presence of ROS. Briefly, cells treated with GA were harvested and rinsed with PBS, mixed with CM-H2DCFDA at a final concentration of 2μM for 30 min at 37 degrees, washed twice with PBS and resuspended in PBS, and flow cytometry was performed immediately using 488-nm excitation with 508/20-nm emission filters using the FACS Calibur (BD Biosciences, Heidelberg, Germany). Intracellular ROS was calculated as the fluorescence intensity.

### Measurement of mitochondrial membrane potential (ΔΨm)

Mitochondrial membrane potential (ΔΨm) induced by GA was measured by microscopic imaging analysis and FACS with the fluorescent probe JC-1 (Thermo Fisher Scientific, Waltham, MA, USA) and Tetramethylrhodamine methyl ester (TMRM) (Thermo Fisher Scientific, Waltham, MA, USA), respectively. For imaging analysis, log-phase cells plated in 24 well plates were treated with GA (500nM) for various times and then incubated with JC-1 for 30 min at a final concentration of 200nM. Images were captured using a Carl Zeiss LSM780 microscope equipped with an EC Plan-Neoflour 10x NA objective and showed with 10X zoom. For FACS analysis, GA (500nM) treated cells were trypsinized and labelled with TMRM at a final concentration of 200uM for 30 min at 37 degrees. Stained cells were analyzed using 488-nm excitation with 573-nm emission filters using a FACS Calibur (BD Biosciences, Heidelberg, Germany). Mitochondrial membrane potential was quantified based on decreases in red fluorescence intensity.

### NADPH/NADP^+^ measurements

NADPH, NADP^+^ production was analyzed with the NADPH/NADP^+^ Glo assay kit (Promega, Madison, WI) according to the manufacture's protocol. Briefly, 4000 cells were seeded in a 96-well plate and incubated for 24H, then cells were treated with GA 500nM for 1and 2 hours before lysing with 1% DTAB. Cell extracts were divided into two groups and exposed to either 0.4N HCl or heated at 60°C to eliminate NADP^+^ or NADPH, respectively. Equal volumes of NADP/NADPH-Glo™ detection reagent were added to each well, incubated for 30–60 minutes at room temperature, and NADP^+^ and NADPH were measured separately using a luminometer. The ratio of NADPH and NADP^+^ was then calculated.

### Thioredoxin reductase (Trx R) activity assay

TrxR activity was determined using a Thioredoxin Reductase Colorimetric Assay Kit (Cayman, Ann Arbor, MI). Briefly, cells were incubated with GA 500nM for 1and 2H, washed with PBS and homogenized in assay buffer. The supernatant was collected after centrifugation, and equal volumes of total protein were added into a 96-well plate with a master mix containing DTNB and NADPH. Absorbance at 405nm during the initial 15min was recorded in a spectrophotometer. TrxR activity was calculated using the formula provided by the protocol.

### Thioredoxin (Trx) activity assay

The Trx activity was determined using a Fluorescent Thioredoxin Activity Assay Kit (Cayman, Ann Arbor, MI, USA) with insulin as the substrate. PCAP-1 cells were treated with GA (500nM) for 1 and 2H, and LuCaP organoids cells were treated with GA (1μM) for 12 and 24H. LuCaP cells were harvested following dispase treatment to dissolve the matrigel. After treatment, cells were washed with PBS and homogenized in assay buffer. Equal volumes of cell lysate were incubated with the substrate in the presence of excess NADPH and TrxR for 30 mins. Fluorescence intensity was recorded for a period of 1H at 545nm with 520nm excitation using an Infinite 200PRO multimode reader (Tecan, Männedorf, Switzerland). Trx activity was calculated using the formula provided by the protocol.

### Western blots

Whole Cell lysates were harvested with RIPA buffer (Boston BioProducts, Ashland, MA, USA) with protease and phosphatase inhibitors. Western blot detection was carried out using SuperSignal™ West Femto Maximum Sensitivity Substrate (Thermo Fisher Scientific, Waltham, MA, USA). Membranes were stripped and blotted with α-tublin as a loading control.

### Statistical analysis

All the results and data were confirmed in at least three separate experiments and presented as mean ± SE. Prizm software (GraphPad Software, Inc.) was used for data analysis. Differences among individual groups were determined by Student's t-test or one-way analysis of variance (ANOVA) followed by Bonferroni's post test for comparisons among 3 or more groups. P < 0.05 was considered statistically significant.

## SUPPLEMENTARY MATERIALS FIGURE AND TABLES



## Abbreviations

PrCa: prostate cancer
CRPC: castration resistant prostate cancer
GA: gambogic acid
ROS: reactive oxygen species
ADT: androgen deprivation therapy
AR: androgen receptor
TCM: traditional Chinese medicine
DHT: Dihydrotestosterone
PDX: patient-derived xenograft
PI: propidium iodide
DFOM: deferoxamine mesylate salt, MMP, mitochondrial membrane potential
NAC: N-acetyl-cysteine
AUR: auranofin
TrxR: thioredoxin reductase
BSO: Buthionine sulfoximine
Trx: thioredoxin
Dox: docetaxel
Enza: enzalutamide
CTG: cell titer glo assay
