# Supplementary material for: Gambogic acid inhibits thioredoxin activity and induces ROS-mediated cell death in castration-resistant prostate cancer
Source: Oncotarget. 2017 Aug 24;8(44):77181–94. doi: 10.18632/oncotarget.20424 (PMC5652772; doi:10.18632/oncotarget.20424)
Supplement: Supplementary file 1 [file oncotarget-08-77181-s001.pdf]

Gambogic acid inhibits thioredoxin activity and induces ROS-mediated cell death in castration-resistant prostate cancer

SUPPLEMENTARY MATERIALS

Supplementary Table 1: Selected genomic characteristics of various LuCaPs

LuCaPs	23.1	73	92	136	167
**Source**	Autopsy	Surgery	Autopsy	Surgery	
**Tissue origin**	Lymph node	Prostate	Lymph node	Ascites fluid	Liver metastasis
**Histology**	Adeno	Adeno	Adeno	Adeno	Adeno
Tp53	One copy loss	Somatic mutation	One copy loss, somatic mutation	One copy loss, somatic mutation	Copy neutral
PTEN	Copy neutral	Copy neutral	One copy loss	Two copy loss	One copy loss
AR	Copy neutral	Copy neutral	High copy gain	Copy neutral	High copy gain
Rb1	One copy loss	Copy neutral	Copy neutral	Copy neutral	One copy loss
Brca2	One copy loss	Copy neutral	Copy neutral	Copy neutral	Copy neutral

Adeno=Adenocarcinoma.

Supplementary Table 2: Synergistic analysis of GA and Docetaxel (DOX)

GA(nM)	DOX(nM)	Effected fraction	CI
250.0	1.0	0.776	1.1069
375.0	1.0	0.967	0.64437
500.0	1.0	0.996	0.46716

CI=Combination Index.

Supplementary Table 3: Synergistic analysis of GA and Enzalutamide (ENZA)

GA(nM)	MDV(µM)	Effected fraction	CI
250.0	10.0	0.802	1.0472
375.0	10.0	0.9549	0.67584
500.0	10.0	0.9951	0.55502

CI=Combination Index.

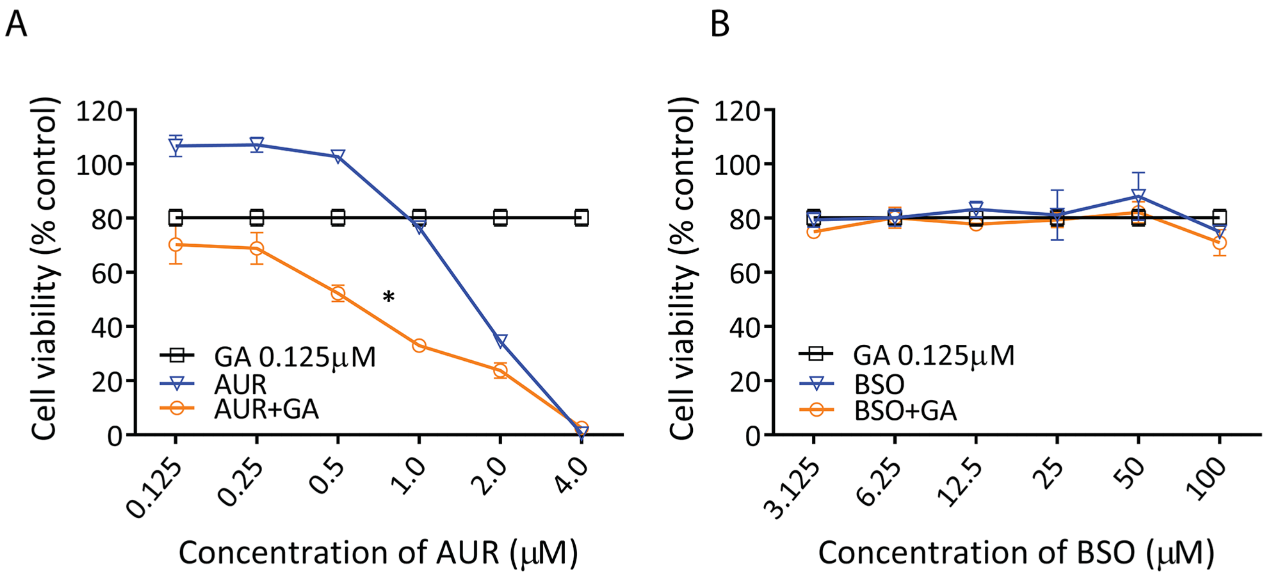

Supplementary Figure 1: (A-B) Viability of PCAP-1 cell treated with GA in combination with various concentration of AUR(A) and BSO(B) for 24h by CTG assay. Results shown here are the average of three independent experiments. *P<0.05, **P<0.01, vs the untreated group.
